# UDP‐Glucose‐6‐Dehydrogenase Mediated O‐GlcNAcylation of Tight Junction Protein 1 Suppresses Metastasis in Renal Cell Carcinoma

**DOI:** 10.1002/advs.202522640

**Published:** 2026-08-19

**Authors:** Xiaolin Chen, Yuhang Bao, Tao Liu, Jianhui Qiu, Yuanyuan Ma, Zedan Zhang, Chuandong Wang, Ruiyi Deng, Zheng Song, Tianyi He, Haode Liu, Jingcheng Zhou, Yizhou Wang, Kan Gong

**Affiliations:** ^1^ Department of Urology Peking University First Hospital Beijing China; ^2^ Institute of Urology Peking University Beijing China; ^3^ Beijing Key Laboratory of Precision Medicine and Innovative Translation for Urogenital Diseases Beijing China; ^4^ National Urological Cancer Center Beijing China; ^5^ National Cancer Center National Clinical Research Center for Cancer Cancer Hospital Chinese Academy of Medical Sciences Peking Union Medical College Beijing China; ^6^ Laboratory Animal Center Peking University First Hospital Beijing China; ^7^ Department of Central Laboratory Peking University First Hospital Beijing China

**Keywords:** metastasis, O‐GlcNAcylation, renal cell carcinoma, TJP1, UGDH

## Abstract

Tumor metastasis remains the leading cause of postoperative recurrence and mortality in patients with clear cell renal cell carcinoma (ccRCC). Although aberrant glucuronic acid metabolism and O‐GlcNAcylation are closely associated with tumor progression, the mechanism by which these pathways intersect to regulate ccRCC metastasis remains poorly understood. Here, an orthotopic ccRCC mouse model coupled with comparative proteomic analysis of primary and metastatic lesions identified UDP‐glucose‐6‐dehydrogenase (UGDH) as a pivotal suppressor of metastasis in ccRCC. In vitro and in vivo functional assays confirmed that UGDH overexpression markedly suppressed the invasion and migration of ccRCC cells. Mechanistically, we demonstrated that the E3 ubiquitin ligase TRIM25 facilitates UGDH degradation via K48‐linked polyubiquitination, whereas UGDH modulates the expression of tight junction protein 1 (TJP1) via O‐GlcNAcylation. Notably, pharmacological inhibition of O‐GlcNAcylation with OSMI‐1 effectively impeded metastasis in both cellular and animal models. This study reveals a novel tumor‐suppressive role for UGDH and highlights the potential of the TRIM25–UGDH–TJP1 axis for diagnostic and therapeutic strategies in metastatic ccRCC.

## Graphical abstract

Our research unveiled a regulatory paradigm wherein TRIM25 orchestrates the ubiquitin‐mediated degradation of UGDH. UGDH modulates the protein stability of TJP1 by regulating O‐GlcNAcylation levels, effectively impeding the metastasis of ccRCC. Our insights elevate UGDH to a pivotal biomarker and tumor suppressor, marking the first demonstration that UGDH depletion activates the O‐GlcNAcylation pathway. This breakthrough delineates a novel therapeutic strategy for ccRCC metastasis, leveraging the inhibition of OGT via OSMI‐1.

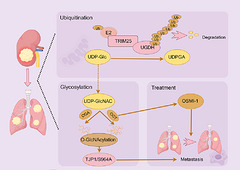

## 1 Introduction

Renal cell carcinoma (RCC) is one of the three most prevalent malignant neoplasms of the urinary system. Clear cell RCC (ccRCC), the most common and aggressive histological subtype, is characterized by a high propensity for metastasis, which represents the leading cause of ccRCC‐related mortality [1, 2]. The lungs, bones, and liver are the most frequently documented sites of metastasis. However, the molecular mechanisms driving the spread of malignant cells are not fully understood [3, 4]. Furthermore, metastatic ccRCC shows considerable resistance to conventional radiotherapy and chemotherapy. This therapeutic challenge and the absence of reliable biomarkers highlight the need to understand the disease's molecular basis [5, 6]. Identifying novel early prognostic biomarkers and elucidating pertinent therapeutic targets is particularly vital for facilitating early detection and improving clinical management [7, 8, 9, 10].

Carbohydrate metabolism, particularly glucose metabolism, plays pivotal roles in cancer cells metabolic reprogramming, particularly tumor initiation, progression, immune evasion, and therapeutic resistance [11, 12]. This metabolic pathway not only provides energy and biosynthetic precursors that are essential for cancer cell proliferation but also orchestrates malignant tumor behavior by modulating signaling pathways, epigenetic alterations, and the tumor immune microenvironment [11, 12]. UDP‐glucose‐6‐dehydrogenase (UGDH) is a critical metabolic enzyme that catalyzes the conversion of UDP‐glucose to UDP‐glucuronic acid, a fundamental process in glycosaminoglycan biosynthesis with essential physiological function, including extracellular matrix remodeling [13]. UDP‐glucose is indispensable for glycosylation reactions and one of the most ubiquitous glycosyl donors [14, 15, 16], acting as both a direct glycosyl donor and frequent precursor, thereby playing a pivotal role in cellular metabolism by bridging carbohydrate metabolic pathways and glycosylation processes [17, 18, 19]. UGDH expression is strongly associated with survival in patients with cancer. Elevated UGDH levels are highly correlated with unfavorable outcomes in lung adenocarcinoma, whereas reduced UGDH levels are associated with poor prognosis in the C4/differentiated subtype of ovarian cancer [13, 20]. Differential expression patterns observed across diverse cancer types highlight UGDH as a potential driver and therapeutic target of cancer metastasis [13, 20, 21]. However, the precise function of UGDH in RCC has not been adequately documented.

O‐GlcNAcylation represents a dynamic monosaccharide post‐translational modification involving the addition of β‐N‐acetylglucosamine (O‐linked β‐N‐acetylglucosamine) to serine and threonine residues [22, 23]. This modification is precisely regulated by a complementary enzymatic system; O‐GlcNAc transferase (OGT) facilitates β‐N‐acetylglucosamine addition, whereas O‐GlcNAcase (OGA) catalyzes β‐N‐acetylglucosamine removal. As this modification is modulated by nutritional and stress cues, it has been designated a “nutrient sensor” [22, 24]. O‐GlcNAcylation profoundly influences protein stability, interactions, subcellular localization, and functional activity, exhibiting intricate crosstalk with other modifications such as phosphorylation; consequently, GlcNAcylation governs virtually all cellular processes, including transcription, signaling, metabolism, and stress responses [24, 25]. Notably, O‐GlcNAcylation drives tumor metastasis by regulating oncogenic signaling, metabolic reprogramming, tumor microenvironment remodeling, and therapeutic resistance. Its dynamic and ubiquitous nature makes GlcNAcylation an attractive therapeutic target, particularly for inhibiting metastatic progression and overcoming drug resistance [26, 27, 28, 29]. However, no previous studies have documented the correlation between the UGDH pathway and O‐GlcNAcylation.

In this study, we identified UGDH as a key molecule in ccRCC metastasis through an orthotopic mouse model coupled with proteomic analysis, and demonstrated that UGDH inhibits ccRCC metastasis both in vitro and in vivo. We further demonstrated that TRIM25 interacts with UGDH and facilitates its degradation via ubiquitination, whereas UGDH modulates TJP1 expression via O‐GlcNAcylation, thereby repressing RCC metastasis. Additionally, mutation of the O‐GlcNAcylation site at S964 in TJP1 effectively impedes RCC metastasis. The OGT inhibitor OSMI‐ 1 blocks metastasis, revealing the role of the TRIM25–UGDH–TJP1 axis and highlighting these molecules as potential biomarkers for ccRCC staging, grading, diagnosis, and monitoring.

## 2 Results

### 2.1 UGDH is Identified as a Metastasis‐Associated Molecule via Proteomic Analysis

**FIGURE 1:**
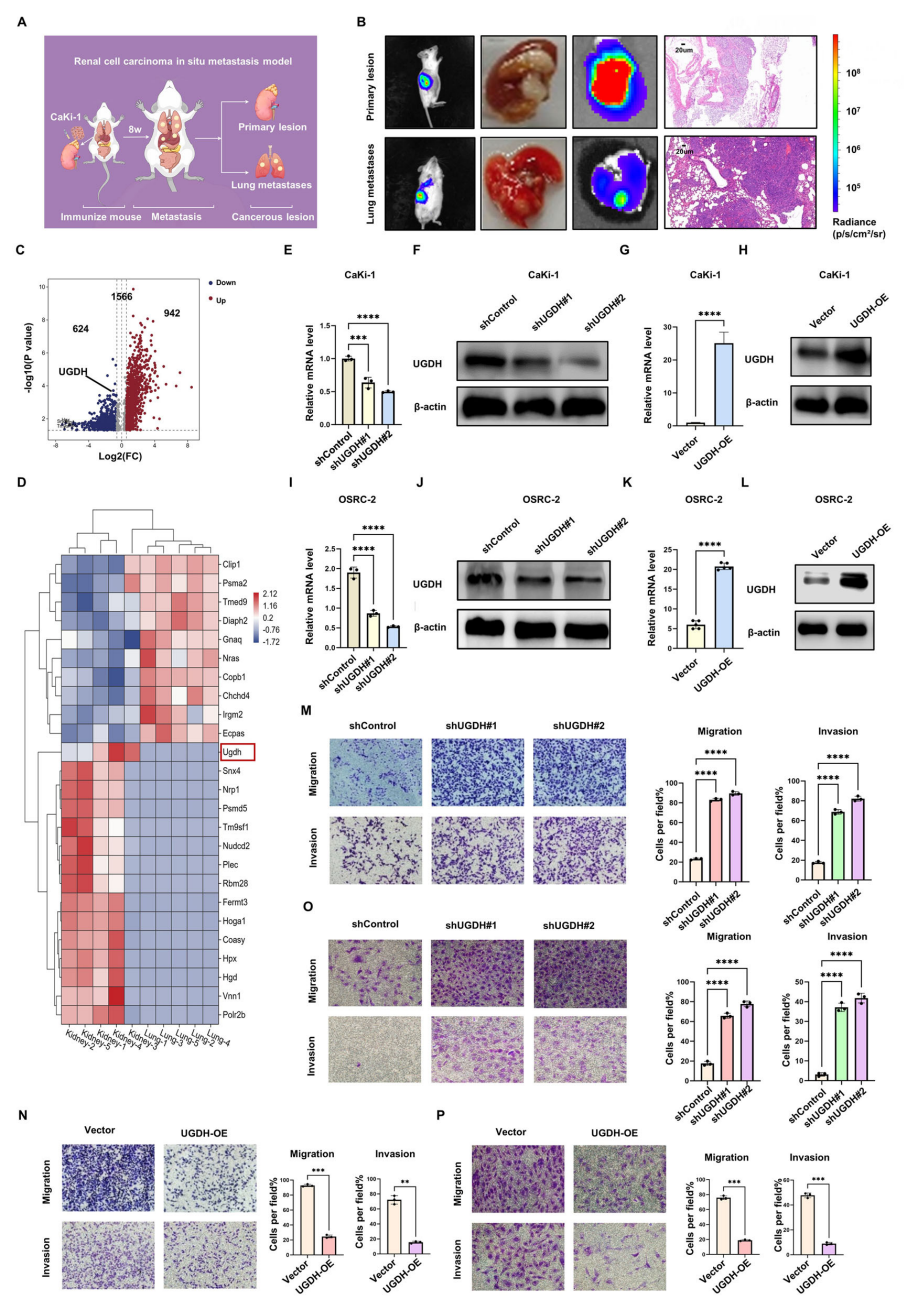
(A) Schematic representation of the orthotopic metastasis model for renal carcinoma (RCC) in murine subjects. (B) In vivo and ex vivo bioluminescent imaging, along with hematoxylin and eosin (H&E) staining, depicting lung metastases within the orthotopic metastasis murine model [*n* = 5]. (C) Volcano plot illustrating differentially expressed molecules identified through proteomic sequencing analysis of primary RCC lesions and their corresponding lung metastases. Significance criteria:|log_2_FC|>0.5 and a nominal *p* < 0.05 were highlighted. (D) Heatmap showcasing co‐expressed differentially expressed molecules from proteomic sequencing analysis of primary RCC lesions and their paired lung metastases in murine subjects. Hierarchical clustering was applied for visualization. *n=5, two‐tailed paired Student's t‐test*. (E–H) Quantitative PCR (qPCR) and western blot validation outcomes for UGDH knockdown and overexpression in CaKi‐1 cells. (I–L) qPCR and western blot validation results for UGDH knockdown and overexpression in OSRC‐2 cells. (M–N) Transwell migration and invasion assays following UGDH knockdown and overexpression in CaKi‐1 cells. (O,P) Transwell migration and invasion assays following UGDH knockdown and overexpression in OSRC‐2 cells. Data in E, I, M, and O were analyzed using one‐way ANOVA followed by Dunnett’s multiple‐comparisons test, with shControl as the reference group. Data in G, K, N, and P were analyzed using two‐tailed unpaired Student’s t‐tests.

To systematically identify the key molecules implicated in ccRCC metastasis, we established a murine orthotopic transplantation model using the CaKi‐1 RCC cell line (Figure 1A,B). Proteomic analysis of matched primary tumors and lung metastases from five mice revealed substantial differences in protein expression profiles. Specifically, compared to primary tumors, 942 proteins were upregulated and 624 were downregulated in metastatic lesions, underscoring significant proteomic distinctions between sites (Figure 1C). Notably, UGDH, a critical metabolic enzyme that catalyzes the conversion of UDP‐glucose to UDP‐glucuronic acid, exhibited significantly lower expression in metastases than in primary tumors, suggesting a potential role as a metastasis suppressor (Figure 1C,D). UGDH exerts both pro‐ and anti‐tumorigenic effects in various cancer types by modulating tumor microenvironment, cancer stem cell function, and drug resistance [20, 30, 31]. However, the functional significance and mechanistic role of UGDH in RCC, particularly during metastasis, remain poorly understood.

Therefore, to investigate the function of UGDH in ccRCC metastasis, we generated stable UGDH‐knockdown and UGDH‐overexpression models using two human ccRCC cell lines, CaKi‐1 (Figure 1E–H) and OSRC‐2 (Figure 1I–L). We then assessed the impact of UGDH modulation on cell migration and invasion using Transwell assays. In CaKi‐1 cells, UGDH knockdown significantly enhanced the migratory and invasive capabilities, whereas UGDH overexpression effectively suppressed these processes (Figure 1M,N). These metastasis‐promoting and metastasis‐inhibiting phenotypes were consistently observed in OSRC‐2 cells (Figure 1O,P), confirming that UGDH suppresses metastasis in ccRCC cells.

To determine whether UGDH activity affects RCC metastasis, we performed ELISA‐based enzyme activity assays (standard curve, R^2^ = 0.9996; Figure S1A). The results showed that UGDH enzyme activity did not differ significantly among different RCC cell lines (HK2, OSRC‐2, and CaKi‐1; Figure S1C) or paired clinical tissue samples (normal, primary tumor, and lung metastasis; Figure S1B). These findings suggest that the inhibitory effect of UGDH on ccRCC metastasis is mainly associated with differences in protein expression levels, rather than changes in its intrinsic enzymatic activity.

### 2.2 UGDH Suppresses ccRCC Metastasis In Vivo

**FIGURE 2:**
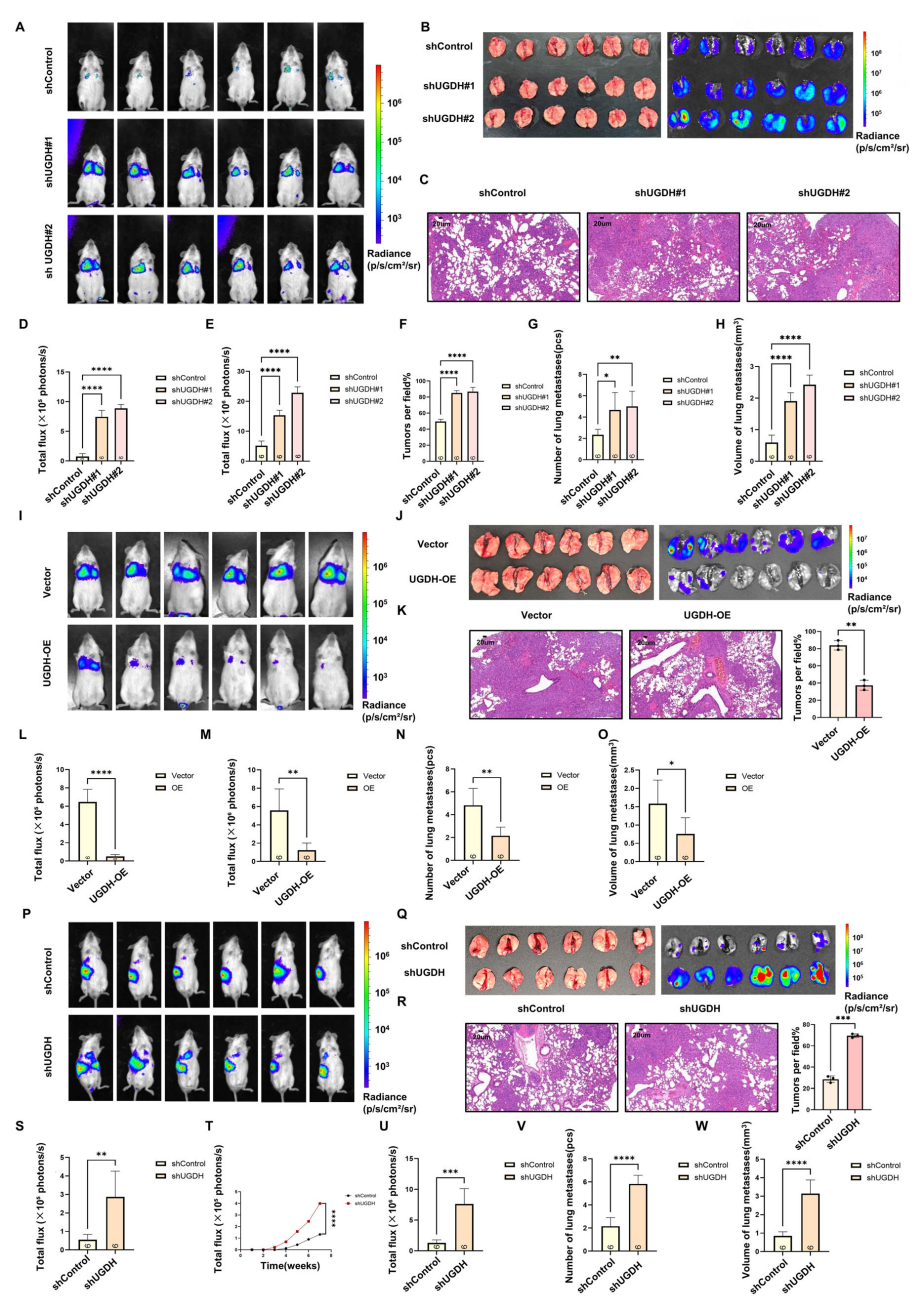
(A–C) In vivo imaging, post‐mortem lung metastasis visualization, and H&E staining outcomes of lung metastases in control and UGDH‐knockdown groups within a tail vein metastasis murine model at endpoint [*n* = 6]. (D–F) Statistical evaluation of the results in A‐C. (G,H) Quantitative analysis of the number and invasive extent of lung metastases in control and UGDH‐knockdown groups within a tail vein metastasis murine model at endpoint. Data in D‐H are presented as the mean ± SEM, *n* = 6, one‐way ANOVA followed by Dunnett’s multiple‐comparisons test, with shControl as the reference group. I–K) In vivo imaging, post‐mortem lung metastasis visualization, and H&E staining outcomes of lung metastases in control and UGDH‐overexpression groups within a tail vein metastasis murine model at endpoint [*n* = 6]. (L,M) Statistical evaluation of the results in (I–K. (N) Quantitative analysis of the number of lung metastases in control and UGDH‐overexpression groups within a tail vein metastasis murine model at endpoint. (O) Quantitative analysis of the invasive extent of lung metastases in control and UGDH‐overexpression groups within a tail vein metastasis murine model at endpoint. (P–R) In vivo imaging, post‐mortem lung metastasis visualization, and H&E staining outcomes of lung metastases in control and UGDH‐knockdown groups within an RCC orthotopic metastasis murine model at endpoint [*n* = 6]. (S,U) Statistical evaluation of the results in (P–R). (T) Statistical line graph depicting in vivo imaging of lung metastases in control and UGDH‐knockdown groups within an RCC orthotopic metastasis murine model over weeks 1–7. Data in L‐O,S and U‐W are presented as the mean ± SEM, *n* = 6, unpaired Student’s t‐test. Data in T were analyzed using repeated‐measures two‐way ANOVA with time, group, and their interaction as factors. (V–W) Quantitative analysis of the number and invasive extent of lung metastases in control and UGDH‐knockdown groups within an RCC orthotopic metastasis murine model at endpoint.

To substantiate the inhibitory effect of UGDH on ccRCC metastasis in vivo, we engineered a murine tail vein metastasis model and an RCC orthotopic transplantation model. In the tail vein model, the UGDH‐knockdown group showed significantly stronger luciferase signals in the lungs than the control group, as evidenced by both in vivo and ex vivo imaging. Moreover, hematoxylin and eosin (H&E) staining of lung tissues revealed a markedly higher tumor burden in the UGDH‐knockdown group than in the control group (Figure 2A–F). In addition, the UGDH‐knockdown group showed a higher number of lung metastatic lesions, larger lesion sizes, and a broader extent of invasion (Figure 2G,H), whereas the UGDH‐overexpression group showed a pronounced inhibitory phenotype across all assessed parameters (Figure 2I–O), indicating that UGDH overexpression effectively suppressed lung metastasis in RCC.

To replicate the metastatic process of RCC cells more accurately, we developed an orthotopic transplantation model using UGDH‐knockdown and control CaKi‐1 cells (Figure S1E,F). In this model, UGDH knockdown significantly increased both in vivo and ex vivo lung tissue luciferase signals compared to those in the control group. H&E staining further confirmed the substantial expansion of the tumor colonization area in the UGDH‐knockdown group (Figure 2P–U). In addition, the UGDH‐knockdown group showed a greater number, size, and invasion range of lung metastatic lesions than the control group (Figure 2V–W). Collectively, our in vitro and in vivo experiments validated the efficacy of UGDH in suppressing ccRCC metastasis.

### 2.3 TRIM25 Regulates UGDH Degradation through Ubiquitination

**FIGURE 3:**
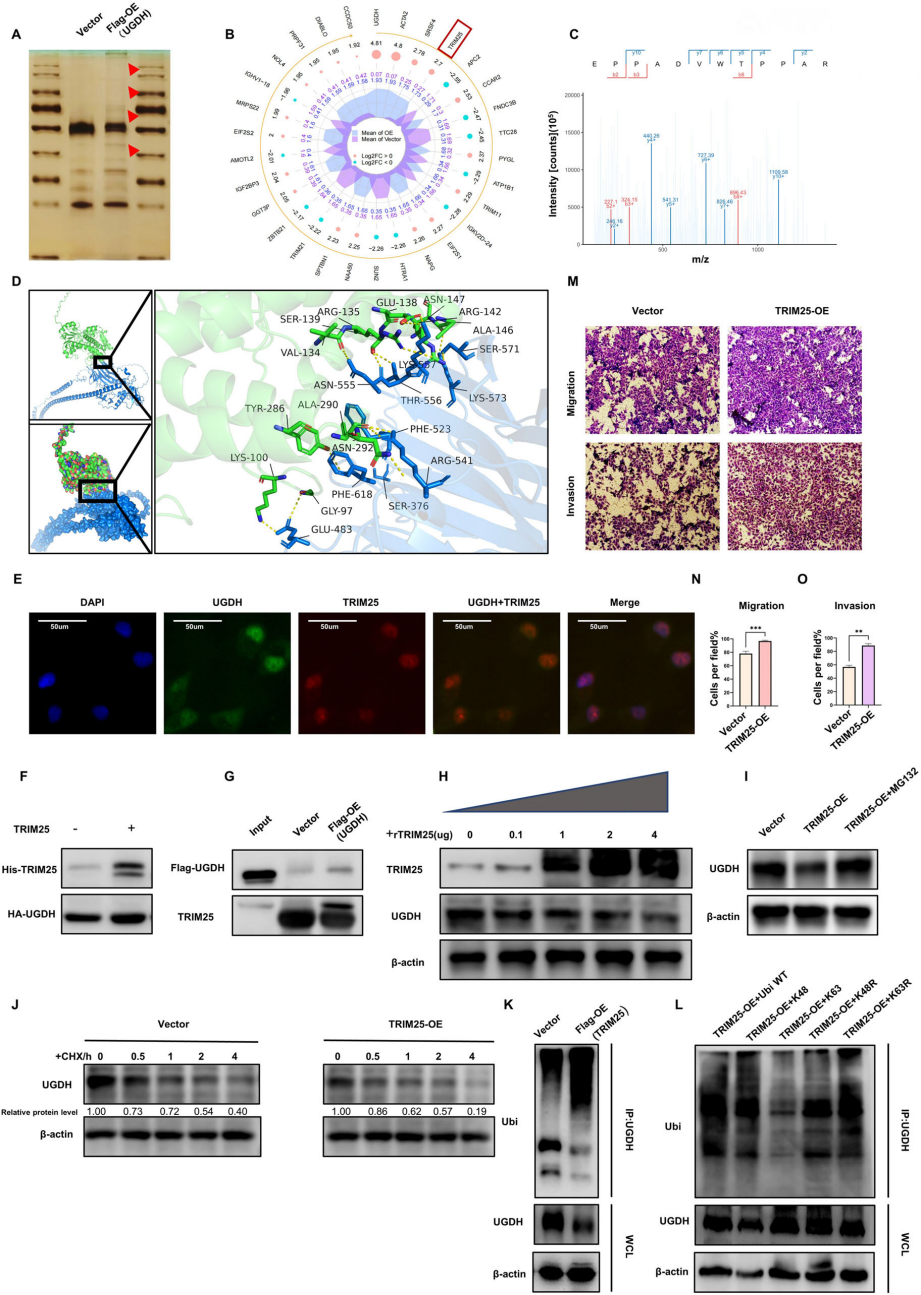
(A) Silver staining prior to immunoprecipitation–mass spectrometry. (B) Top 30 molecules exhibiting significant interaction differences with UGDH protein, as identified by immunoprecipitation–mass spectrometry. (C) Secondary mass spectrometry analysis of TRIM25 peptide fragment. (D) Molecular docking results of TRIM25 and UGDH protein. (UGDH: pdb_00003tdk; TRIM25:pdb_00009iun). (E) Fluorescent co‐localization of UGDH and TRIM25. F) In vitro binding (pull‐down) assay showing the direct interaction between purified TRIM25 and UGDH. Bacterially expressed HA‐tagged UGDH was used as bait to pull down His‐tagged TRIM25. (G) Exogenous co‐immunoprecipitation validation of the TRIM25‐UGDH interaction. (H) Dose‐dependent overexpression of the E3 ubiquitin ligase TRIM25 and the ubiquitination‐induced degradation profile of its substrate protein UGDH. (I) MG132‐mediated inhibition of UGDH ubiquitination and degradation. (J) Cycloheximide chase analysis of UGDH protein stability in vector control and TRIM25‐overexpressing CaKi‐1 cells. UGDH band intensities were normalized to the corresponding β‐actin signals, and the normalized UGDH level at each time point was expressed relative to that of the respective group at 0 h, which was set to 1.00. The relative band intensities from the representative experiment are indicated below the blots.K) UGDH pull‐down ubiquitination assay in TRIM25‐overexpressing cells. (L) Ubiquitination assay showing the ubiquitin linkage type involved in TRIM25‐mediated UGDH ubiquitination. Cell lysates were subjected to immunoprecipitation with an anti‐UGDH antibody, followed by immunoblotting for ubiquitin in the presence of wild‐type, K48‐only, K63‐only, K48R, or K63R ubiquitin constructs. (M–O) Transwell migration and invasion assays and corresponding data analysis in control and TRIM25‐overexpressing groups within CaKi‐1 cells. Data are presented as the mean ± SEM, *n* = 3, independent samples t‐test.

To investigate the upstream regulatory mechanisms of UGDH, we employed immunoprecipitation‐mass spectrometry to screen for proteins interacting with UGDH and successfully identified the E3 ubiquitin ligase TRIM25 (Figure 3A–C). Molecular docking (Figure 3D), immunofluorescence co‐localization (Figure 3E), in vitro binding assays (Figure 3F), and exogenous co‐immunoprecipitation (Figure 3G) confirmed the interaction between TRIM25 and UGDH. Further investigation revealed that TRIM25 overexpression markedly decreased UGDH protein levels in a dose‐dependent manner (Figure 3H). Treatment of TRIM25‐overexpressing cells with the proteasome inhibitor MG132 substantially increased UGDH protein levels, indicating that TRIM25‐mediated UGDH degradation is a proteasome‐dependent process (Figure 3I). In addition, cycloheximide treatment at various time points demonstrated lower stability of the UGDH protein in TRIM25‐overexpressing cells than in control cells (Figure 3J). Immunoprecipitation showed a significant increase in UGDH ubiquitination in TRIM25‐overexpressing cells (Figure 3K). To further characterize the ubiquitin chain topology, we performed immunoprecipitation of UGDH, and compared the ubiquitination patterns among wild‐type, K48‐only, K63‐only, K48R, and K63R ubiquitin constructs. The results showed that UGDH ubiquitination mainly depends on K48‐related chain linkages, whereas the contribution of K63‐related chains linkages was relatively limited. These findings indicate that TRIM25‐mediated ubiquitination of UGDH is predominantly K48‐linked, which is consistent with previously observed proteasome‐dependent degradation (Figure 3L). Finally, TRIM25 overexpression in ccRCC CaKi‐1 cells significantly enhanced their migration and invasion capabilities (Figure 3M–O).

### 2.4 UGDH Knockdown Activates the O‐GlcNAcylation Modification Pathway

**FIGURE 4:**
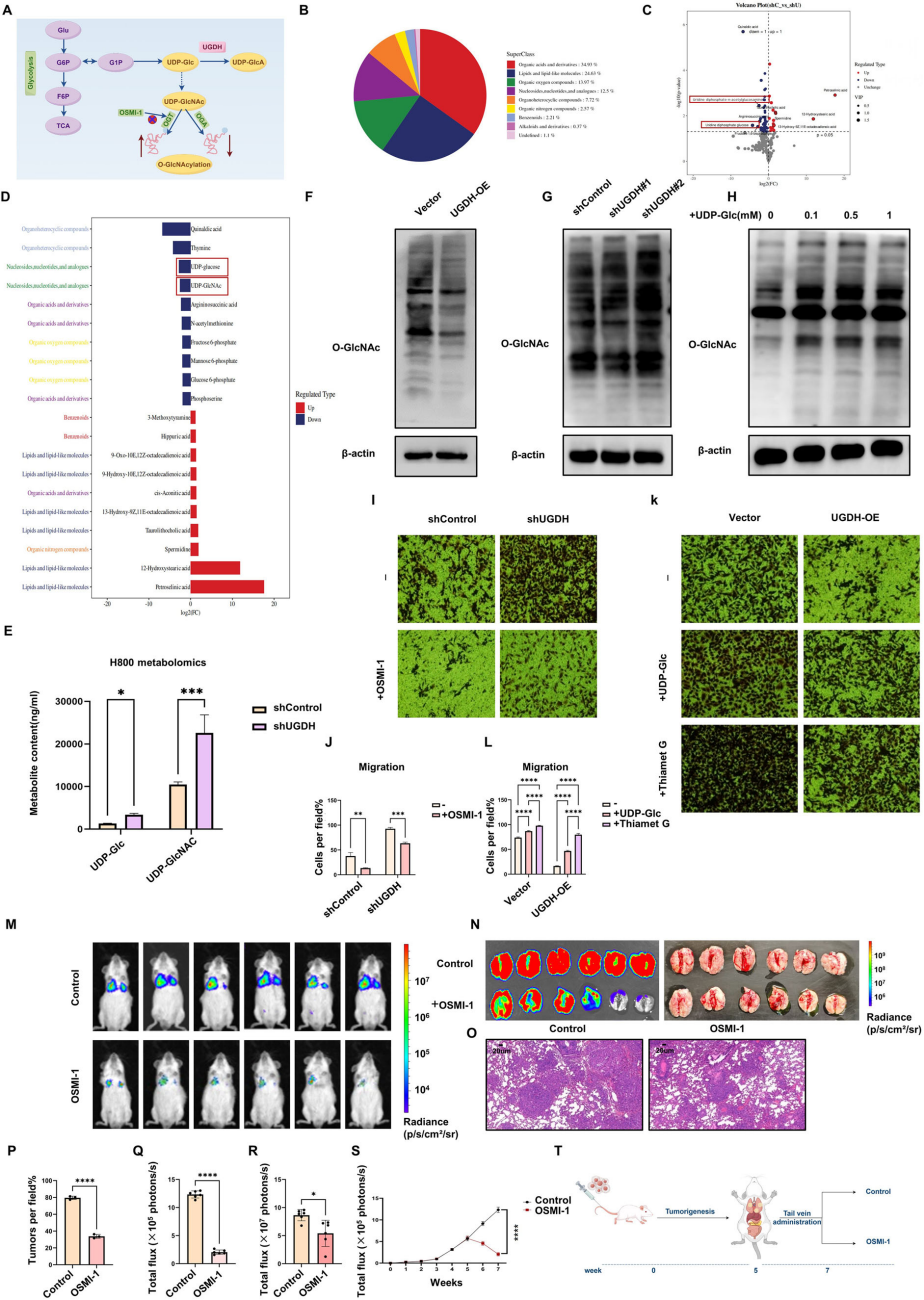
(A) Schematic illustration of the UGDH‐mediated O‐GlcNAcylation pathway. (B) Categorization of differential metabolites identified through targeted metabolomics sequencing in control versus UGDH‐knockdown groups. (C,D) Volcano and butterfly plots depicting candidate differential metabolites from targeted metabolomics analysis in the control and UGDH‐knockdown groups. (E) Quantitative analysis of UDP‐glucose and UDP‐GlcNAc levels derived from targeted metabolomics sequencing in control and UGDH‐knockdown groups [data are presented as the mean ± SEM, *n* = 3, two‐tailed independent samples t‐test]. (F,G) Western blot analysis of O‐GlcNAcylation levels in UGDH‐overexpressing (UGDH‐OE) and UGDH‐knockdown (shUGDH) cells. H) Western blot demonstrating enhanced O‐GlcNAcylation upon UDP‐glucose supplementation. (I,J) Transwell assays revealing the effects of OGT inhibitor (OSMI‐1) on RCC migration in control and UGDH‐knockdown groups [data are presented as the mean ± SEM, *n* = 3, two‐way ANOVA]. (K,L) Transwell assays showing the effects of UDP‐glucose and OGA inhibitor (Thiamet G) on RCC migration in control and UGDH‐overexpression groups [data are presented as mean ± SEM, *n* = 3, two‐way ANOVA]. M–R) In vivo imaging of mice at the seven‐week endpoint after tail vein injection, followed by dissection to visualize lung metastases, H&E staining, and statistical analysis [*n* = 6]. (S) Temporal imaging analysis of lung metastasis progression throughout establishment and treatment phases of the mouse tail vein lung metastasis model [data are presented as the mean ± SEM, *n* = 6, repeated‐measures two‐way ANOVA was used to analyze the effects of time, group, and their interaction]. (T) Diagram of the mouse tail vein lung metastasis model and therapeutic intervention. Mice were administered OSMI‐1 via tail vein injection at a dose of 10 mg/kg every two days for two weeks.

Previous studies have demonstrated that UGDH knockout elevates UDP‐glucose levels in breast cancer cells, with UDP‐glucose serving as the principal donor for glycosylation modifications [20, 30, 31]. Therefore, we investigated whether the UGDH pathway influences RCC metastasis via O‐GlcNAcylation by constructing the relevant pathway diagrams (Figure 4A). Initially, we performed targeted metabolomic sequencing on control and UGDH‐knockdown cells, which revealed significantly higher UDP‐glucose and UDP‐GlcNAc levels in the UGDH‐knockdown group than in the control group (Figure 4B–E). Subsequently, we assessed O‐GlcNAcylation levels in UGDH‐knockdown and UGDH‐overexpression cells and found that O‐GlcNAcylation decreased markedly with UGDH overexpression but increased following UGDH knockdown (Figure 4F,G).

The relationship between UDP‐glucose and UDP‐GlcNAc cannot be inferred solely from changes in steady‐state metabolite levels. Therefore, we supplemented our study with isotope tracing experiments and targeted metabolomic analyses to directly determine whether metabolic flux was redirected after UGDH loss and to clarify the source of increased UDP‐glucose and UDP‐GlcNAc levels.

First, we performed an isotope tracing analysis to examine the isotopic distributions of UDP‐D‐glucose and UDP‐N‐acetyl‐glucosamine.UGDH knockdown altered the isotopolog distribution of UDP‐glucose and UDP‐GlcNAc, with increases in selected labeled fractions. Specifically, the M+3 fraction of UDP‐glucose (Figure S2), as well as the M+2, M+3, M+5, and M+8 fractions of UDP‐N‐acetylglucosamine (Figure S3), showed significant upregulation. These findings indicate that UGDH downregulation does not merely cause passive accumulation of UDP‐glucose but is also accompanied by further flux of its carbon source into the UDP‐GlcNAc biosynthetic pathway. This suggests that the metabolic flux was redistributed, providing evidence for UGDH knockdown enhancing the metabolic connection between UDP‐glucose and UDP‐GlcNAc.

Furthermore, we introduced FR054, an inhibitor of the hexosamine biosynthetic pathway (HBP), and performed targeted metabolomics and western blot analyses. The results showed that FR054 treatment markedly reduced global O‐GlcNAcylation levels, and both UDP‐glucose and UDP‐N‐acetyl‐glucosamine were consistently identified as significantly decreased metabolites (Figure S4B–F). In addition, rescue experiments in UGDH‐knockdown CaKi‐1 cells demonstrated that FR054 treatment significantly suppressed the migratory ability induced by UGDH depletion (Figure S4G). As UDP‐GlcNAc is the direct donor for O‐GlcNAcylation, these results confirm the biological consequences of the UDP‐GlcNAc increase caused by UGDH loss, which ultimately leads to enhanced O‐GlcNAcylation and promotion of the metastatic phenotype. Together, these findings provide strong evidence that the upstream glucose‐derived carbon fluxes of UDP‐glucose and UDP‐GlcNAc are redirected by HBP.

Furthermore, adding varying concentrations of UDP‐glucose to ccRCC cells led to a notable increase in O‐GlcNAcylation levels (Figure 4H). Additionally, we examined O‐GlcNAcylation in common ccRCC cell lines and observed higher glycosylation levels in the metastatic CaKi‐1 strain (Figure S5A). To elucidate the impact of O‐GlcNAcylation on ccRCC metastasis, we introduced the OGT inhibitor (OSMI‐1) and OGA inhibitor (Thiamet G) into ccRCC cells. OSMI‐1 addition significantly inhibited cell migration, indicating that reducing O‐GlcNAcylation suppressed ccRCC metastasis (Figure 4I,J). Conversely, the addition of UDP‐glucose and Thiamet G markedly promoted cell migration, suggesting that elevated O‐GlcNAcylation promotes ccRCC metastasis (Figure 4K,L).

To validate the in vivo inhibitory effect of OSMI‐ 1 on ccRCC metastasis, we constructed a mouse tail vein lung metastasis model (Figure 4T and Figure S5D,E). OSMI‐ 1 was dissolved in DMSO, diluted in PBS, and administered via tail vein injection at a dose of 10 mg/kg every two days for two weeks, starting at six weeks post‐inoculation. The control group received an equivalent volume of vehicle (DMSO/PBS). Compared with mice in the control group, those in the OSMI‐ 1 treatment group had weaker luciferase signals in both in vivo and ex vivo lung metastasis lesions at the endpoint. Additionally, H&E staining showed that the tumor burden in the OSMI‐ 1 group was significantly lower than that in the control group (Figure 4M–R). In vivo bioluminescence imaging was performed weekly, whereas endpoint analyses (ex vivo imaging and H&E staining) were performed at Week 7 (Figure 4S). In summary, both in vitro and in vivo experiments confirmed that O‐GlcNAcylation modification promotes RCC metastasis, UGDH knockdown promotes RCC metastasis by increasing O‐GlcNAcylation levels, and OSMI‐1 represents an effective therapeutic agent for targeting this pathway.

### 2.5 UGDH Regulates TJP1 Expression via O‐GlcNAcylation

**FIGURE 5:**
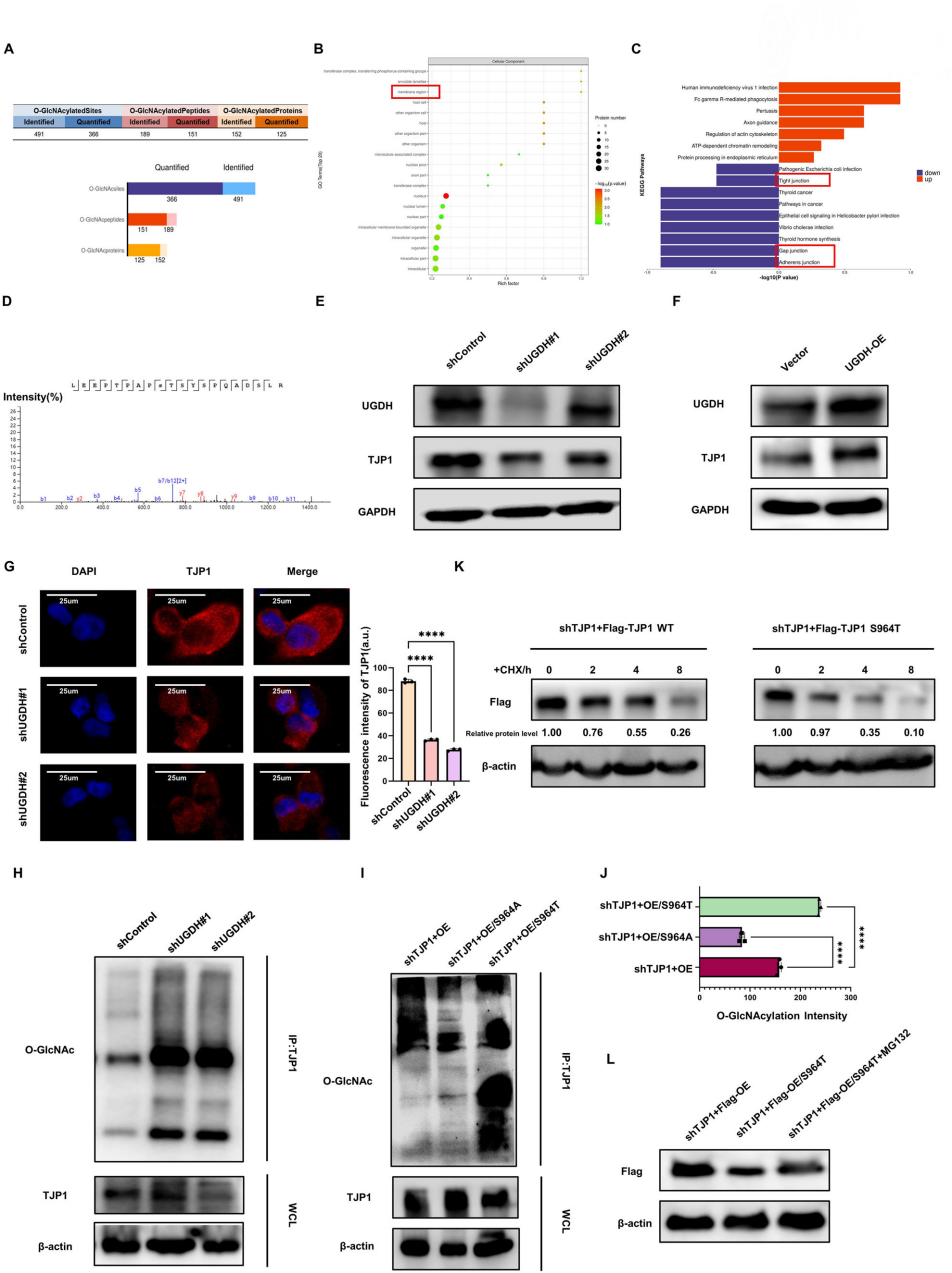
(A) Summary of the O‐GlcNAcylation modification proteomic dataset obtained from shControl and shUGDH CaKi‐1 cells [*n* = 1]. (B,C) Proteomic analysis of O‐GlcNAcylation modification identified tight junction protein 1 (TJP1) through GO and KEGG enrichment. (D) Schematic representation of the O‐GlcNAcylation site S964 on TJP1. (E,F) Western blotting confirmation of UGDH knockdown and overexpression, demonstrating reduced and elevated TJP1 protein levels, respectively. (G) Immunofluorescent visualization of TJP1 expression in control and shUGDH groups. (H) Immunoprecipitation with an anti‐TJP1 antibody revealed enhanced O‐GlcNAcylation in control and UGDH‐knockdown cells. I) TJP1 pull‐down O‐GlcNAcylation assay in shTJP1+OE, shTJP1+OE/S964A, and shTJP1+OE/S964T cells. (J) Statistical analysis of O‐GlcNAcylation modification intensity derived from the TJP1 pull‐down O‐GlcNAcylation assay (three groups: shTJP1+OE, shTJP1+OE/S964A, shTJP1+OE/S964T) [data are presented as the mean ± SEM, *n* = 3, one‐way ANOVA followed by Dunnett's post‐hoc test]. (K) Cycloheximide chase analysis of Flag‐TJP1 protein stability in cells reconstituted with Flag‐TJP1 WT or Flag‐TJP1 S964T. Flag‐TJP1 band intensities were normalized to the corresponding β‐actin signals, and the normalized Flag‐TJP1 level at each time point was expressed relative to that of the respective group at 0 h, which was set to 1.00. The relative band intensities from the representative experiment are indicated below the blots.  (L) MG132‐mediated rescue of TJP1 protein levels, confirming proteasome‐dependent degradation.

To elucidate the precise downstream molecular mechanisms by which UGDH modulates O‐GlcNAcylation, we conducted O‐GlcNAcylation proteomic analyses on RCC cell lines harboring short hairpin control (shControl) or UGDH‐knockdown (shUGDH) cells. Sequencing revealed 152 O‐GlcNAcylation‐associated proteins, 189 peptide segments, and 491 modification sites, including 125 proteins with 151 quantifiable O‐GlcNAcylated peptide segments and 366 quantifiable modification sites (Figure 5A). KEGG and GO analyses identified tight junction protein 1 (TJP1), which is intricately linked to metastasis (Figure 5B,C), along with its specific O‐GlcNAcylation site (S964) (Figure 5D). To corroborate the regulatory nexus between UGDH and TJP1, western blot and immunofluorescence assays demonstrated significantly reduced TJP1 expression in the UGDH‐knockdown group and markedly increase TJP1 expression in the UGDH‐overexpression group (Figure 5E–G).

Subsequently, to ascertain whether UGDH governed TJP1 via O‐GlcNAcylation, immunoprecipitation experiments confirmed the enhanced TJP1 binding to O‐GlcNAcylation in the UGDH‐knockdown group (Figure 5H). To further investigate the mechanism by which O‐GlcNAcylation at the TJP1 S964 site regulates protein expression, immunoprecipitation and O‐GlcNAcylation‐focused mass spectrometry revealed that the S964A mutation markedly reduced the O‐GlcNAcylation signal of TJP1, whereas the S964T mutant exhibited a stronger O‐GlcNAcylation signal. This indicates that S964 is a key site regulating the O‐GlcNAcylation status of TJP1 (Figure 5I,J). We further performed cycloheximide chase assays, which showed that the TJP1 protein was degraded markdly faster in the S964T reconstitution group than in the wild‐type reconstitution group under the same conditions (Figure 5K). In addition, the cells were treated with the proteasome inhibitor MG132, which significantly restored TJP1 protein levels when TJP1 expression was reduced, indicating that TJP1 loss was dependent on the proteasomal degradation pathway (Figure 5L).

Additionally, Transwell assays verified that TJP1 knockdown promoted ccRCC cell migration and invasion, whereas TJP1 overexpression on a TJP1‐knockdown background significantly impeded these processes. Mutation of the TJP1 O‐GlcNAcylation site S964 to S964 to S964A further suppressed ccRCC metastasis (Figure S7D,E). TJP1, a classical tight junction scaffold protein, should be further evaluated for its role in maintaining cell–cell junction integrity, epithelial barrier function, and paracellular permeability. At the level of functional adhesion and cell–cell junctions, shTJP1 cells exhibited a looser cellular arrangement and weaker intercellular contacts than shControl cells, suggesting that TJP1 downregulation disrupts the integrity of cell–cell adhesion (Figure S7G). Furthermore, we performed FITC‐dextran permeability assay, which demonstrated that TJP1 reduction substantially alters the paracellular permeability and junctional integrity of the cell layer, contributing to RCC metastasis (Figure S7H). Consequently, we deduced that UGDH sustains TJP1 protein stability by modulating O‐GlcNAcylation levels and that TJP1, particularly the TJP1‐S964A O‐GlcNAcylation site, effectively inhibits RCC metastasis.

### 2.6 Expression of TRIM25/UGDH/TJP1 in Renal Primary Tumor, Adjacent Tissues, and Corresponding Metastatic Lesions

**FIGURE 6:**
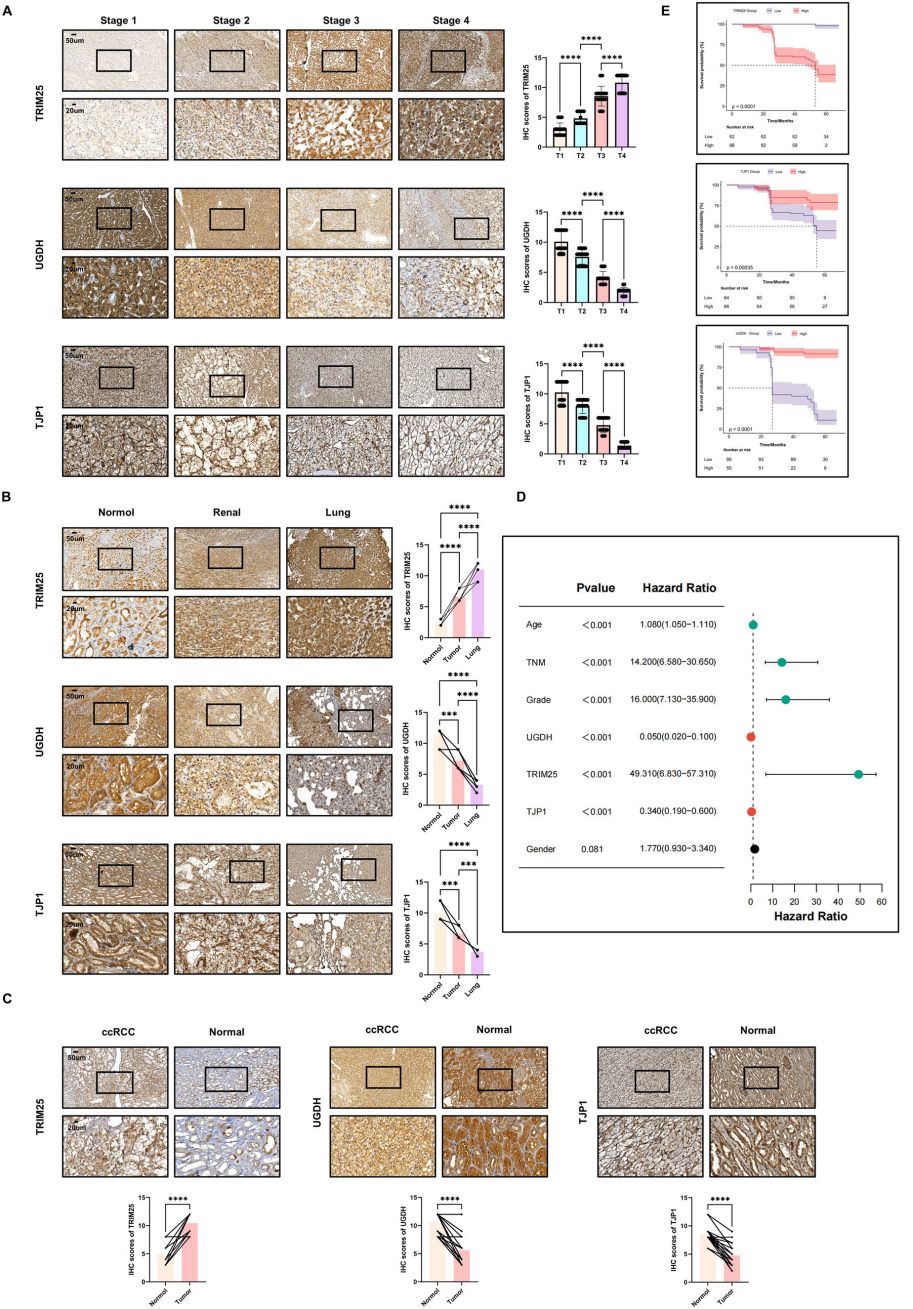
(A) Histopathological examination of renal clear cell carcinoma across various stages: T1 (*n* = 50), T2 (*n* = 50), T3 (*n* = 25), and T4 (*n* = **25**), accompanied by immunohistochemical findings and statistical evaluation [data are presented as the mean ± SEM, *n* = 150, one‐way ANOVA]. (B) Comparative immunohistochemical and statistical analysis of RCC, adjacent tissues, and corresponding lung metastases in matched cases [data are presented as the mean ± SEM, *n* = 10 matched cases, and were analyzed using repeated‐measures one‐way ANOVA followed by Tukey’s multiple‐comparisons test]. (C) Immunohistochemical assessment and statistical analysis of RCC and adjacent tissues in a cohort of 150 patients with RCC [data are presented as the mean ± SEM, *n* = 150, paired Student's t‐test]. (D) Forest plot showing multivariate Cox proportional hazards regression analysis of clinicopathological variables and protein expression levels in patients with ccRCC. Age, TNM stage, grade, UGDH, TRIM25, TJP1, and sex were included in the model. Hazard ratios with 95% confidence intervals are presented to evaluate independent prognostic significance [*n* = 150, Cox regression analysis]. (E) Kaplan–Meier survival analyses of patients with ccRCC stratified by TRIM25, UGDH, and TJP1 expression levels. Patients with high TRIM25 expression exhibited significantly poorer overall survival, whereas high UGDH and TJP1 expression were associated with better overall survival [*n* = 150, log‐rank test].

To elucidate the role of the TRIM25–UGDH–TJP1 axis in ccRCC and contextualize our findings within a clinicopathological framework, we evaluated the protein expression levels of TRIM25, UGDH, and TJP1 in ccRCC specimens via immunohistochemical analysis. Pathological sections were obtained from 150 patients diagnosed with ccRCC spanning stages T1 (*n* = 50), T2 (*n* = 50), T3 (*n* = 25), and T4 (*n* = 25), along with sections from 10 patients with paired lung metastases. Our findings revealed a positive correlation between TRIM25 expression and tumor histological staging and grading, whereas UGDH and TJP1 expression levels were negatively correlated with tumor staging and grading (Figure 6A and Figure S8A). In paired samples, TRIM25 expression was markedly elevated in both primary and metastatic renal tumors, whereas UGDH and TJP1 expression was significantly reduced. These observations are consistent with our quantitative proteomic analyses of primary and metastatic RCC lesions in murine models (Figure 6B,C and Figure S8B).

This analysis was supplemented with prognostic analyses. Kaplan–Meier survival curves showed that high TRIM25 expression was significantly associated with poor survival, whereas high UGDH and TJP1 expression was associated with better prognosis (Figure 6E). According to further Cox regression analyses, after adjusting for clinical variables, including age, TNM stage, grade, and sex, UGDH, TRIM25, and TJP1 remained statistically significant, indicating that these markers possess independent prognostic relevance. In particular, UGDH was identified as a protective factor, TRIM25 as a risk factor, and TJP1 likewise as a protective factor (Figure 6D).

In conclusion, our data suggest that TRIM25, UGDH, and TJP1 represent novel biomarkers for the pathological staging and grading of ccRCC and early detection of metastasis.

## 3 Discussion

Metastasis of RCC drives patient mortality and postoperative recurrence, with metabolic reprogramming—particularly glucose metabolism—playing a pivotal role in tumor development and progression [1, 2, 4, 32, 33]. Consequently, identifying novel metabolic driver genes and elucidating the molecular mechanisms by which they modulate tumor growth are vital for developing targeted therapies and improving patient prognoses. In this study, we used a murine orthotopic transplantation model and subjected primary renal tumors and lung metastases to proteomic sequencing, which revealed significant downregulation of UGDH in lung metastases. This downregulation was correlated with adverse clinicopathological features and poor prognosis, indicating that UGDH may serve as a novel biomarker for ccRCC. Further experimentation demonstrated that UGDH markedly inhibited the migration and invasion of ccRCC cells in vitro; this inhibitory effect was confirmed in vivo using both tail vein and orthotopic metastasis models. The intrinsic catalytic activity of UGDH did not differ significantly across cell lines with varying metastatic potential or between clinical tissue types, indicating that the metastasis‐associated function of UGDH is driven by altered protein abundance rather than changes in enzymatic activity. Our study is the first to establish UGDH as a tumor suppressor in RCC that effectively impedes ccRCC metastasis.

Ubiquitination is a pivotal post‐translational protein modification in tumor metastasis, modulating protein stability, functionality, localization, and interactions [34, 35, 36, 37, 38, 39]. This process governs proteostatic balance, dictating protein degradation or stabilization through the interplay of diverse E3 ubiquitin ligases and deubiquitinating enzymes, thereby directly influencing tumor cell migration and invasiveness [11, 40, 41]. To elucidate the upstream regulators of UGDH, we employed immunoprecipitation–mass spectrometry analysis, which revealed direct interactions between UGDH and several proteins, and identified the E3 ligase TRIM25 as a key upstream regulatory protein. In glioblastoma, TRIM25 promotes metastasis by facilitating K63‐linked ubiquitination of the NONO protein, whereas in prostate cancer, TRIM25 drives drug resistance and metastasis via the ubiquitination of the androgen receptor [40, 42, 43]. Through an in vitro binding assay, co‐immunoprecipitation, immunofluorescence co‐localization, and molecular docking, we confirmed previous research reporting the UGDH–TRIM25 interaction and demonstrated that TRIM25 promotes UGDH protein degradation via K48‐linked ubiquitination, enhancing the migration and invasion of ccRCC cells. Although convergent evidence from multiple approaches strongly supports TRIM25 as a mediator of UGDH ubiquitination, a fully reconstituted in vitro ubiquitination system and systematic domain mapping do not yet exist. Molecular docking analysis suggested that the interaction interface may involve the SPRY domain of TRIM25; however, this finding requires further experimental validation.

O‐GlcNAcylation functions as a pivotal nexus in the intricate interplay between metabolism and epigenetics, dynamically modulating a multitude of proteins to harmonize nutritional cues with cellular processes [22]. O‐GlcNAcylation dysregulation is profoundly implicated in metabolic disorders, neuropathies, and cancer progression; thus, targeting of OGT/OGA or downstream effector proteins represents a burgeoning therapeutic paradigm [22, 25, 26]. Considering that UGDH knockout elevates UDP‐glucose, a ubiquitous donor for glycosylation processes, changes in UDP‐glucose levels may indirectly influence UDP‐GlcNAc concentrations by perturbing overall glucose metabolism or the HBP, potentially impacting O‐GlcNAcylation [30, 44, 45].To corroborate this hypothesis, we employed targeted metabolomics, ^1^ ^3^C‐glucose isotope tracing, and western blot analyses, which revealed that UGDH knockdown augmented levels of the direct donor UDP‐GlcNAc and UDP‐glucose, and enhanced O‐GlcNAcylation. Isotope tracing demonstrated that the metabolic flux was redirected through the HBP after UGDH loss, providing mechanistic evidence beyond mere correlative metabolite changes. Treatment with the HBP inhibitor (FR054) reversed the enhanced migration of shUGDH cells, providing functional validation. In vitro assays demonstrated that UDP‐glucose supplementation promoted ccRCC cell migration, which was exacerbated by enhanced O‐GlcNAcylation. As well as providing the first evidence for UGDH knockdown activating the O‐GlcNAcylation pathway, our in vitro and in vivo experiments confirm the potential of the OGT inhibitor OSMI‐ 1 as a therapeutic agent for RCC.

O‐GlcNAcylation proteomic analysis identified TJP1 as a downstream effector and S964 as a key O‐GlcNAcylation site. We validated that UGDH governs TJP1 protein expression through O‐GlcNAcylation, thereby influencing RCC metastasis. The S964A mutation markedly reduced TJP1 O‐GlcNAcylation and suppressed migration, whereas the S964T mutant enhanced O‐GlcNAcylation and weakened migration suppression. Cycloheximide chase assays demonstrated that the S964T mutant exhibited accelerated degradation compared to wild‐type TJP1, and that MG132 rescued proteasome‐dependent degradation. Furthermore, TJP1 knockdown impaired cell–cell adhesion, increased Vimentin expression, and enhanced paracellular permeability, underscoring the functional role of TJP1 in epithelial integrity. These results support a model in which O‐GlcNAcylation at S964 modulates TJP1 protein stability through the proteasomal pathway.

Although several combination therapies have been approved for advanced ccRCC, the molecular biomarkers necessary for guiding therapeutic decisions are lacking [44]. Robust molecular biomarkers are pivotal in managing tumor metastasis, particularly for staging, grading, and early diagnosis [46, 47, 48, 49, 50]. To translate our findings into a clinically relevant framework, we examined the protein levels of TRIM25, UGDH, and TJP1 via immunohistochemical staining in a cohort comprising 150 ccRCC patient pathological sections, including paired lung metastatic sections from 10 patients. Our findings revealed significant upregulation of TRIM25 in both primary and metastatic ccRCC samples, whereas UGDH and TJP1 expression were markedly downregulated. Multivariate Cox regression adjusted for age, TNM stage, grade, and sex identified UGDH and TJP1 as independent protective factors and TRIM25 as a risk factor. In summary, our data suggest that TRIM25/UGDH/TJP1 is a novel biomarker for the pathological staging and early detection of ccRCC metastasis.

This study has some limitations that should be acknowledged. First, although two independent pharmacological inhibitors (OSMI‐1 targeting OGT and FR054 targeting HBP) reversed the pro‐migratory phenotype of UGDH‐deficient cells, genetic OGT depletion (siRNA/shRNA) with concurrent assessment of TJP1 stability was not performed, which would provide definitive mechanistic confirmation and is a priority for future work. Second, the cycloheximide chase experiment compared wild‐type TJP1 and S964T only; inclusion of the S964A mutant in the cycloheximide chase assay would further strengthen the evidence chain linking O‐GlcNAcylation status at S964 to protein stability. Third, although our molecular docking analysis suggests that the SPRY domain of TRIM25 may mediate interactions with UGDH, systematic domain truncation and rescue experiments have not yet been completed. Fourth, a fully reconstituted in vitro ubiquitination assay (using recombinant E1, E2, E3, ubiquitin, and substrate) for the TRIM25–UGDH axis has not yet been established. Fifth, we did not perform detailed co‐immunoprecipitation with other tight junction components (e.g., claudins and occludin), subcellular fractionation experiments, systematic cytoskeletal reorganization analysis, cell–matrix adhesion assays, or trans‐epithelial electrical resistance measurements, which would further clarify the downstream cellular mechanisms of TJP1‐mediated metastasis suppression. Sixth, independent prospective validation of the prognostic value of TRIM25/UGDH/TJP1 in a separate cohort would enhance the translational relevance of our findings. Future endeavors may also entail developing therapeutic strategies to restore UGDH expression in preclinical ccRCC models—via exosome‐ or nanoprobe‐mediated delivery systems—and conducting clinical trials to validate the therapeutic efficacy of the O‐GlcNAcylation inhibitor (OSMI‐1) in patients with ccRCC. Additionally, whether feedback inhibition on upstream HBP enzymes (GFAT, GNPNAT, PGM3, and UAP1) contributes to the observed metabolic flux redistribution remains an open question.

**FIGURE 7:**
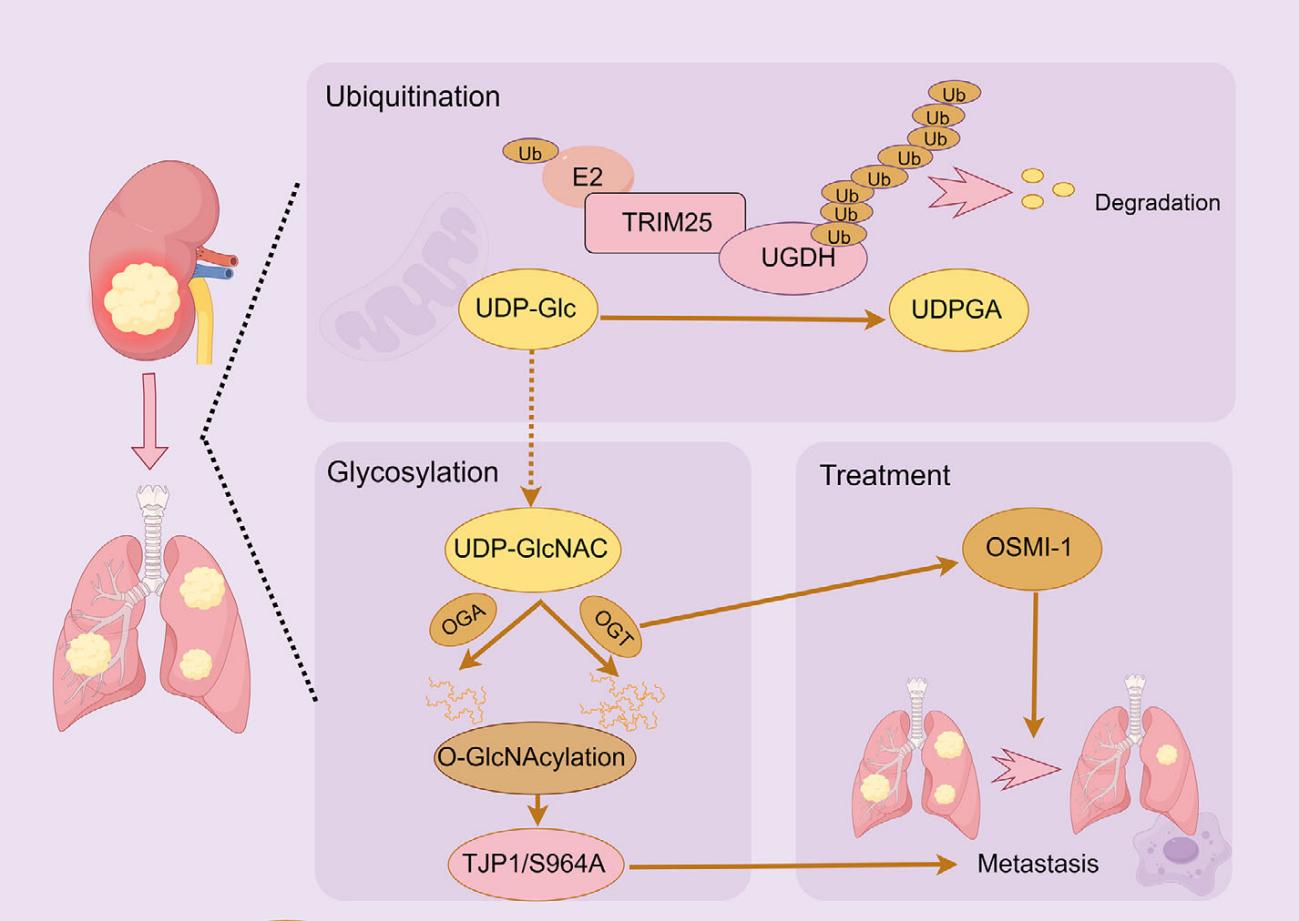
Schematic model demonstrating that the E3 ubiquitin ligase TRIM25 facilitates UGDH degradation via K48‐linked ubiquitination, whereas UGDH modulates the stability of the tight junction protein TJP1 via O‐GlcNAcylation.

In conclusion, our study revealed a regulatory paradigm in which TRIM25 orchestrates the ubiquitin‐mediated degradation of UGDH (Figure 7). UGDH modulates the protein stability of TJP1 by regulating O‐GlcNAcylation levels, effectively impeding ccRCC metastasis. Our insights elevate UGDH as a pivotal biomarker and tumor suppressor and demonstrate for the first time that UGDH depletion activates the O‐GlcNAcylation pathway. This breakthrough highlights a novel therapeutic strategy for ccRCC metastasis that leverages OGT inhibition via OSMI‐ 1.

## 4 Experimental Section

### 4.1 Ethical Statement

This study was conducted in accordance with the Declaration of Helsinki and was approved by the Institutional Ethics Committee of Peking University First Hospital (approval No.2025‐ 1489). Written informed consent was obtained from all participants or their legally authorized representatives. All animal experimental procedures were approved by the Experimental Animal Ethics Committee of Peking University First Hospital (approval No. J2025140).

### 4.2 Sample Collection and Patient Follow‐up in Clear Cell Renal Cell Carcinoma

A multicenter cohort of 150 patients with ccRCC was established. Tissue microarrays containing 80 paired tumor and adjacent normal tissue samples were purchased from Shanghai Outdo Biotech (YP‐KicSur2201, China). Tumor specimens and adjacent non‐neoplastic tissues were collected from 70 patients who underwent surgical resection for ccRCC at Peking University First Hospital between January 2020 and August 2025. Among these, 10 had matched pulmonary metastatic samples. Comprehensive clinicopathological parameters, including patient demographics (age and sex), tumor grade, TNM classification, and five‐year follow‐up data, were systematically compiled for subsequent analyses. The prognostic significance of UGDH, TRIM25, and TJP1 expression in relation to patient outcomes was evaluated using Kaplan–Meier survival analysis and multivariate Cox regression.

### 4.3 Cell Culture

Human ccRCC cell lines, including CaKi‐1, OSRC‐2, and 293T cells for lentivirus packaging, were obtained from Pricella (PC‐H2023013140, PC‐H2023013134, PC‐H2025090303, China, 2023/06/07), all of which survived normally without contamination. 786‐O and OSRC‐2 cells were cultured in RPMI‐ 1640 medium (Gibco, USA), and other cell lines were maintained in DMEM (Gibco, USA). All media were supplemented with 10% fetal bovine serum (Gibco, USA) and 1% penicillin‐streptomycin (Gibco, USA). Cells were maintained in a humidified incubator at 37°C with 5% CO_2_.

### 4.4 Lentiviral Packaging and Infection

Lentiviral packaging was performed in 293T cells using a three‐plasmid system (target plasmid: psPAX2: pMD2.G = 2:1:1) following transfection with Lipofectamine 3000 (Thermo Fisher Scientific, USA). Target cells were transduced with the lentiviral particles and selected using puromycin or G418 to establish stable knockdown or overexpression of the target gene. RNA and protein were extracted to validate the transduction efficiency.

### 4.5 Cell Migration and Invasion Assays

After overnight serum starvation, 1–5 × 10^4^ cells were seeded into the upper chamber of a Transwell insert (Corning 3422, USA) coated with basement membrane matrix gel (Beijing Langli Biotechnology Co., China) for invasion assays, or left uncoated for migration assays. DMEM containing 10% FBS was added to the lower chamber and serum‐free DMEM was placed in the upper chamber. To inhibit proliferation, 100 nM of 10058‐F4 (MedChemExpress, USA) was added to the medium (IC_50_ = 99.58 nM in CaKi‐1 cells; Figure S1D). After incubation for 24 h, the cells were fixed with 4% paraformaldehyde, stained with 0.1% crystal violet then counted using a microscope. Cells per field% represents the percentage of tumor cells in each field of view in figures.

### 4.6 RNA Extraction and Quantitative PCR

Total RNA was extracted from the cells using TRIzol reagent (Invitrogen, USA) according to the manufacturer's instructions. Complementary DNA was synthesized using the M‐MLV Reverse Transcription Kit (Invitrogen, USA). qPCR was performed using SYBR Green PCR premix (Roche) on the AriaMx Real‐Time PCR System (Agilent Technologies, USA). Gene expression levels were normalized to GAPDH as an internal control, and relative expression was calculated using the 2^−ΔΔCt method. The primer sequences used for qPCR are listed in Table S1.

### 4.7 Western Blot Analysis and Antibodies

Protein extracts from tissues and cells were prepared using pre‐chilled RIPA lysis buffer (Beyotime, China) supplemented with protease and phosphatase inhibitors. Protein concentrations were determined using the BCA protein assay kit (Thermo Fisher Scientific, USA). Samples were denatured at 95°C for 10 min, separated by SDS‐PAGE, and transferred onto PVDF membranes for immunoblotting. The primary antibodies used in this study are listed in Table S2.

### 4.8 Immunohistochemistry and Immunofluorescence Analysis

Immunohistochemistry was performed on paraffin‐embedded ccRCC tissue sections. After deparaffinization, rehydration, and blocking, the sections were incubated with primary antibodies against target proteins. The immunohistochemistry score was calculated as the product of the staining‐intensity score (0 = negative, 1 = weak, 2 = moderate, 3 = strong) and the positive cell proportion score (0 = negative, 1 = 1%–25%, 2 = 26%–50%, 3 = 51%–75%, 4 = 76%–100%). Sections with a score ≥6 were defined as the high‐expression group. For immunofluorescence analysis, cells were fixed and permeabilized, then incubated with primary antibodies at 4°C overnight, followed by fluorescent secondary antibodies at 37°C for 2 h in the dark. Nuclei were counterstained with DAPI for 10 min and images were acquired using a confocal microscope.

### 4.9 H&E Staining

Tumor and adjacent tissue specimens were fixed in 4% paraformaldehyde overnight, embedded in paraffin, and sectioned into 4 µm‐thick slices. H&E staining was performed to evaluate pulmonary metastatic burden in xenograft mice.

### 4.10 Co‐Immunoprecipitation

For transfection‐based co‐immunoprecipitation assays, proteins were extracted from renal cancer cells transfected with plasmids expressing Flag‐tagged constructs. The lysates were incubated with anti‐Flag antibodies and protein A/G agarose beads (Beyotime, China) at 4°C overnight. After three washes with lysis buffer, the immunocomplexes were eluted and analyzed using western blotting. For endogenous co‐immunoprecipitation, ccRCC cell lysates were incubated at 4°C overnight with specific primary antibodies or normal IgG as a control, followed by incubation with protein A/G agarose beads. The immunoprecipitated complexes were washed, eluted, and a small aliquot of the eluate was mixed with SDS sample buffer for western blotting, with the remaining portion stored at −80°C.

### 4.11 Affinity Purification–Mass Spectrometry (IP‐MS) Analysis

Samples were thawed from −80°C to room temperature, and protein concentrations were determined using a BCA protein assay kit (Thermo Fisher Scientific, USA). Proteins were reduced with DTT, alkylated with IAA, and digested with trypsin overnight at 37°C. Peptides were separated on an EASY‐nLC 1200 ultra‐high‐performance liquid chromatography system with gradient elution and analyzed on an Orbitrap Exploris 480 mass spectrometer in data‐independent acquisition mode, optimized for high‐resolution tandem mass spectrometry acquisition.

### 4.12 Luciferase Reporter Assay

ccRCC cells were seeded in six‐well plates and grown to approximately 80% confluence before transfection with plasmids containing a luciferase reporter construct. After 48 h of incubation, luciferase activity was measured following the manufacturer's instructions. D‐luciferin potassium salt (Lablead, China) was added as a substrate, and luminescence intensity was recorded immediately.

### 4.13 Orthotopic Tumor Progression in RCC

The animal experiments were conducted in accordance with the institutional guidelines for animal welfare and experimental management. Six‐week‐old B‐NDG mice (Biocytogen, China) were used to establish an orthotopic renal tumor xenograft model. Approximately 1×10^6^ luciferase‐expressing cells were suspended in 20 µl medium containing 2% calf serum and injected into the renal capsule of the mice. At 13 weeks of age, the mice underwent in vivo bioluminescent imaging for tumor assessment, followed by dissection. For imaging, the mice were anesthetized and administered 150 mg/kg D‐luciferin via intraperitoneal injection, then positioned laterally or supine in an IVIS‐50 chamber (Caliper Life Sciences, USA). Images were processed using Living Image software (Caliper Life Sciences, USA). Bioluminescence signals within the regions of interest were quantified as background‐subtracted total flux and expressed as photons per second (photons/s). Pseudocolor images were displayed as radiance (photons/s/cm²/sr), with identical minimum and maximum radiance scales applied to all animals within the same experiment.

### 4.14 Establishment of a Renal Cancer Metastasis Model via Tail Vein Injection

An experimental metastasis model was established by injecting 1×10^6^ human renal cancer CaKi‐1 cells into the tail veins of five‐week‐old B‐NDG mice (Biocytogen, China). For the OSMI‐1 treatment model, CaKi‐1 cells were injected via the tail vein on day 0. OSMI‐1 (10 mg/kg, dissolved in DMSO and diluted in PBS) was administered via tail vein injection every two days starting from six week for two weeks. The control group received an equivalent volume of vehicle (DMSO/PBS). In vivo bioluminescence imaging was performed weekly, and endpoint analyses, including ex vivo imaging and H&E staining, were performed at Week seven.

### 4.15 Evaluation of Pulmonary Metastasis

After dissection, the lungs were placed in an IVIS‐50 chamber (Caliper Life Sciences, USA). Bioluminescence signals were captured, and images were processed with Living Image software (Caliper Life Sciences, USA). Regions of interest encompassing the lung‐associated bioluminescent signals were defined, and the signals were quantified as background‐subtracted total flux and expressed as photons per second (photons/s). Pseudocolor images were displayed as radiance (photons/s/cm²/sr), with identical minimum and maximum radiance scales applied to all groups within the same experiment. The number of bioluminescent metastases and lung surface area (measured under bright‐field conditions) were used to calculate pulmonary metastatic burden, expressed as the ratio of metastatic area to total lung surface area. Patients were stratified into high‐ and low‐expression groups according to the prespecified IHC score cutoff for each marker.Lung metastatic volume (mm³) was calculated as Volume = (length × width²) / 2.

Lungs were then fixed in 4% formaldehyde, embedded in paraffin, and sectioned to a thickness of 5 µm. Metastatic lesions were identified by H&E staining. Thirty lung sections per mouse were randomly selected for analysis. Metastatic burden was assessed microscopically (Leica CME, Leica Microsystems, Cambridge, UK) based on lesion size, with foci containing <5, 6–15, or >15 cells assigned scores of 1, 2, or 3, respectively. The metastatic index was defined as the sum of these scores. For metastatic foci spanning consecutive sections, only the largest cross section was scored. Scoring was independently performed by two researchers blinded to group allocation.

### 4.16 Glycosylomics

For protein extraction, cells were lysed in 8 M urea buffer containing 100 mM Tris/HCl (pH 8.5). Protein concentrations were determined using a BCA protein assay kit. For SDS‐PAGE, equal amounts of protein (15 µg per sample) were denatured, separated on 4%–20% SDS‐PAGE gradient gels, and stained with Coomassie Brilliant Blue. For in‐Solution Digestion, proteins were reduced with 10 mM DTT, alkylated with 20 mM IAA, diluted to lower urea concentration, and digested with trypsin overnight at 37°C. The resulting peptides were adjusted to neutral pH, desalted, and lyophilized. Then, O‐GlcNAcylated peptide were enrichment by incubating the samples with anti‐GlcNAc‐S/T affinity beads, followed by thorough washing and elution with TFA. The eluates were desalted before liquid chromatography–tandem mass spectrometry analysis. Then, peptides were separated using a NanoElute HPLC system and analyzed on a timsTOF Pro mass spectrometer operated in parallel accumulation‐serial fragmentation mode with active exclusion protocols.

Two glycosylation proteomic experiments were conducted. The first dataset (shControl and shUGDH) was generated from a single sample (*n* = 1) and used primarily for discovery purposes. The second dataset (IP‐TJP1:shTJP1+OE, shTJP1+OE/S964A, shTJP1+OE/S964T), which included biological replicates (*n* = 3), showed acceptable reproducibility across samples. In both analyses, peptide and protein identification were controlled at a 1% false discovery rate at both the peptide‐spectrum match and protein levels, and O‐GlcNAcylation sites identificaton was considered robust when the site localization probability exceeded 0.75.

### 4.17 Proteomics

Tissue samples were homogenized in T‐PER buffer and proteins were precipitated with acetone, centrifuged, and air‐dried. The dried pellets were dissolved in urea, reduced with TCEP, alkylated with IAA, diluted, and digested with trypsin overnight at 37°C. The resulting peptides were desalted, dried, reconstituted in formic acid, and analyzed by nano‐liquid chromatography‐tandem mass spectrometry using an EASY‐nLC 1200 system coupled to a Q Exactive HF‐X mass spectrometer. Peptide separation was performed on a C18 analytical column in data‐independent acquisition mode with predefined MS1 and MS2 parameters. The data were processed in Spectronaut software and searched against the mouse UniProt database, with carbamidomethylation (C) as a fixed modification and acetylation (protein N‐terminus) and oxidation (M) as variable modifications.

The proteomic analysis included five biological replicates per group (*n* = 5). Protein identification was filtered using a 1% false discovery rate at peptide and protein levels. The Pearson correlation coefficients between biological replicates were >0.90, confirming high reproducibility.

### 4.18 H800 Targeted Metabolomics

The samples were thawed at 4°C and mixed with methanol/acetonitrile/water solution (2:2:1). After vortexing and 30 min of ultrasonication, the samples were incubated at −20°C for 10 min and then centrifuged at 14 000 × g for 20 min at 4°C. The supernatants were lyophilized, reconstituted in acetonitrile/water (1:1), vortexed, and centrifuged for 15 min. Ultra‐high performance liquid chromatography was performed using an Agilent 1290 Infinity LC system equipped with either HILIC or C18 columns. Mass spectrometric analysis was carried out on an AB Sciex 6500+ QTRAP system using electrospray ionization with the following parameters: ion source temperature 580°C, GS1 45, GS2 60, CUR 35, and IS ± 4500 V. Multiple reaction monitoring mode was used for analyte detection. Data processing was performed using MultiQuant software to calculate peak area ratios and analyte concentrations based on the calibration curves. Rigorous quality control procedures were performed to ensure data accuracy, reproducibility, and integrity.

### 4.19 ELISA Assay

ELISA was performed according to the manufacturer's instructions (ELISA kit for UDP‐Glucose‐6‐Dehydrogenase; Univ, China, SEG938Hu‐96T). Briefly, standards and samples were prepared and added to microplate wells pre‐coated with the corresponding capture antibodies. After washing to remove unbound substances, the detection antibody and enzyme conjugate were sequentially added to form immune complexes. Following an additional washing step, the substrate solution was added for color development and the reaction was terminated by adding a stop solution. The absorbance was measured using a microplate reader, and the concentration of the target molecule in each sample was calculated based on the standard curve.

### 4.20 13C‐Based Stable Isotope Tracing Technique

Cells were subjected to metabolic tracing using ^13^C6‐glucose. The cells were subjected to either UGDH knockdownor an HBP inhibitor (FR054, Selleck, USA, E1275) treatment. After collection, metabolites were extracted with pre‐chilled 80% methanol, and the supernatants were harvested after centrifugation. The extracts were vacuum‐dried and reconstituted in 50% methanol/water. Subsequently, metabolites involved in glycolysis, the tricarboxylic acid cycle, the pentose phosphate pathway, and the HBP were detected by liquid chromatography‐high‐resolution mass spectrometry in tSIM mode. Finally, Skyline and MAVEN were used for peak extraction, integration, and natural isotope correction to evaluate the distribution of ^13^C‐labeled isotopologs and associated metabolic flux changes.

### 4.21 FITC‐Dextran Permeability Assay

The cells were seeded into Transwell inserts and cultured until formation of a confluent monolayer. FITC‐dextran solution was then added to the upper chamber, while fluorescence‐free medium was added to the lower chamber. After incubation for the indicated times, the medium from the lower chamber was collected, and the fluorescence intensity was measured using a microplate reader. Permeability of the cell monolayer was evaluated based on the amount of FITC‐dextran that passed through the monolayer.

### 4.22 Cell Aggregation Assay

Control and TJP1‐knockdown cells were dissociated into a uniform single‐cell suspension. Both groups were transfected with plasmids carrying green fluorescent tags. After adjusting the cell density, the cells were cultured under low‐adhesion conditions to allow for spontaneous aggregation. At the indicated time points, cell aggregates were observed and imaged under a fluorescence microscope. The number, size, and extent of aggregate formation were compared between the two groups to evaluate cell‐cell adhesion and aggregation capacity.

### 4.23 Statistical Analysis

Statistical analysis was performed using GraphPad Prism version 10.1.2 (GraphPad Software, San Diego, CA, USA). The association between clinicopathological characteristics and the expression levels of TRIM25, UGDH, and TJP1 was assessed using the chi‐square test. Normally distributed continuous data were presented as the mean ± standard error of the mean (SEM), and comparisons between two independent groups were performed using a two‐tailed unpaired Student's t‐test. For comparisons involving more than two groups, one‐way or two‐way analysis of variance (ANOVA) was applied, followed by Tukey’s or Šídák’s multiple‐comparisons test as appropriate. For matched or paired data, two‐tailed paired Student's t‐test or repeated‐measures ANOVA was used. For non‐normally distributed data, values were presented as medians (interquartile range).Patients were stratified into high‐ and low‐expression groups according to the prespecified IHC score cutoff for each marker. Survival data were analyzed using the Kaplan–Meier method, and intergroup differences were evaluated using the log‐rank test. Multivariate analysis was performed using Cox proportional hazards regression analysis, adjusted for age, TNM stage, grade, and sex. All statistical tests were two‐sided, and *p* < 0.05 was considered statistically significant (^*^ *p* < 0.05, ^**^ *p* < 0.01, ^***^ *p* < 0.001, ^****^ *p* < 0.0001).

## Author Contributions

K.G, Y.W, J.Z, and X.C conceived the project. K.G, Y.W, J.Z, and X.C designed the experiments; X.C, Y.B, T.L, J.Q, Z.Z, C.W, R.D, Z.S, T.H, and H.L performed experiments and analyzed data; X.C and Y.B performed pathologic analysis; K.G provided ccRCC tumor samples and advised on the clinical correlation analysis. X.C and Y.W wrote the manuscript.

## Conflicts of Interest

The authors declare no conflicts of interest.

## Supporting information




**Supporting File 1**: advs76883‐sup‐0001‐SuppMat.docx.

**Supporting File 2**: advs76883‐sup‐0002‐TableS1.xlsx.

**Supporting File 3**: advs76883‐sup‐0003‐TableS2.xlsx.

**Supporting File 4**: advs76883‐sup‐0004‐SuppMat.xlsx.

## Data Availability

The data that support the findings of this study are available from the corresponding author upon reasonable request.
