# Supplementary material for: UDP‐Glucose‐6‐Dehydrogenase Mediated O‐GlcNAcylation of Tight Junction Protein 1 Suppresses Metastasis in Renal Cell Carcinoma
Source: Adv Sci (Weinh). 2026 Aug 19;13(59):e22640. doi: 10.1002/advs.202522640 (PMC13487724; doi:10.1002/advs.202522640)
Supplement: Supplementary file 1 [file ADVS-13-e22640-s001.docx]

**5.Supplementary** **Figures and Figure Legends**

**Figure S1.** A) Standard curve for the ELISA-based UGDH enzyme activity assay. The fitted regression equation and R² value are shown in the plot [data are presented as the mean ± SEM, n=3]. B) UGDH enzyme activity measured in paired normal kidney tissue, primary tumor tissue, and lung metastatic tissue from five patients. No significant differences were observed among the indicated tissue types [data are presented as the mean ± SEM, n=5, repeated-measures one-way ANOVA]. C) UGDH enzyme activity measured in the HK2, OSRC-2, and CaKi-1 cell lines. No significant difference in UGDH enzyme activity was detected among the indicated cell lines [data are presented as mean ± SEM, n=3, one-way ANOVA]. D) Cell viability assay showing the 72-h dose–response curve of 10058-F4 treatment. The calculated IC₅₀ value is indicated in the plot [data are presented as the mean ± SEM, n=5]. E) Representative in vivo bioluminescence images of mice in the shControl and shUGDH groups at an earlier time point after tumor cell inoculation [n=6]. F) Representative in vivo bioluminescence images of mice in the shControl and shUGDH groups at a later time point, showing higher metastatic burden in the shUGDH group than in the shControl group [n=6].

**Figure S2. ¹**³C-isotopolog distribution of UDP-D-glucose in shControl and shUGDH cells. Following ¹³C-glucose isotope tracing, the labeling pattern of UDP-D-glucose was analyzed by metabolomics in shControl and shUGDH cells. The relative fractions of individual isotopologs (M+0 to M+15) are shown. [data are presented as mean ± SEM , n=4. Nominal *P* values were calculated using two-tailed unpaired Student's t-tests; *P* < 0.05].

**Figure S3.** ¹³C-isotopolog distribution of UDP-N-acetyl-glucosamine in shControl and shUGDH cells. After ¹³C-glucose labeling, glucose-derived carbon incorporation into UDP-N-acetyl-glucosamine was analyzed by isotope tracing metabolomics in shControl and shUGDH cells. The relative abundance of each isotopolog (M+0 to M+15) is shown. [data are presented as the mean ± SEM , n=4. Nominal *P* values were calculated using two-tailed unpaired Student's t-tests; *P* < 0.05].

**Figure S4.** A) Metabolomic profiling of the hexosamine biosynthetic pathway (HBP) pathway and effect of the HBP inhibitor FR054 in UGDH-deficient ccRCC cells. Classification of detected metabolites based on the HMDB superclass/category annotation. The number of compounds identified in each metabolite class is shown [data are presented as the mean ± SEM, n=3, independent samples t-test]. B) Heatmap of representative differential metabolites between the UGDH-knockdown (shUGDH) group and its corresponding shUGDH+FR054 group under the indicated conditions. Metabolites are grouped according to HMDB categories, and selected metabolites related to the HBP, including UDP-D-glucose and UDP-N-acetyl-glucosamine, are highlighted. Right-hand panel shows the corresponding log2 fold change (log2FC), VIP values, and −log10(P value) [data are presented as the mean ± SEM, n=3, independent samples t-test]. C) Volcano plot showing significantly altered metabolites between the UGDH-knockdown group and corresponding shUGDH+FR054 group. Upregulated metabolites are shown in red, downregulated metabolites in blue, and unchanged metabolites in gray [data are presented as the mean ± SEM, n=3, independent samples t-test]. D) Heatmap of representative differential metabolites between the control group and shControl+FR054 group. Metabolites are grouped according to HMDB categories, and UDP-D-glucose and UDP-N-acetyl-glucosamine are highlighted. Right-hand panel indicates log2FC, VIP values, and −log10(P value) for each metabolite [data are presented as the mean ± SEM, n=3, independent samples t-test]. E) Volcano plot showing differential metabolite distribution between the control group and shControl+FR054 group. Upregulated metabolites are shown in red, downregulated metabolites in blue, and unchanged metabolites in gray [data are presented as the mean ± SEM, n=3, independent samples t-test]. F) Western blot analysis of global O-GlcNAcylation in cells treated with the HBP inhibitor FR054. FR054 treatment reduced the overall O- GlcNAcylation level from that in the control group. G) Transwell migration assay showing the effect of FR054 on the migratory ability of shUGDH cells. Treatment with FR054 significantly suppressed the enhanced migration induced by UGDH knockdown. Quantification of migrated cells is shown on the right [data are presented as the mean ± SEM, n=3, independent samples t-test]. H) IC₅₀: Cell viability curve of FR054 treatment. Cells were exposed to increasing concentrations of FR054, and viability was measured to determine the inhibitory effect of the compound. The calculated IC₅₀ value is indicated in the plot [data are presented as the mean ± SEM, n=3].

**Figure S5.** A) Expression levels of O-GlcNAcylation in various RCC cell lines. B) Inhibitory effects of the OGT inhibitor OSMI-1 on O-GlcNAcylation at varying concentrations in CaKi- 1 cells. C) Augmentative effects of the OGA inhibitor Thiamet G on O-GlcNAcylation at varying concentrations in CaKi- 1 cells. D–E) Visualization and statistical evaluation of tumor burden in a murine tail vein metastasis model at week 5, immediately prior to treatment initiation [data are presented as the mean+SEM. n=6, independent samples t-test].

**Figure S6.** Evaluation of the cytotoxicity and in vivo safety of OSMI-1. A) Cell viability assay showing the dose–response curve of OSMI-1 treatment. The calculated IC₅₀ value is indicated in the plot [data are presented as the mean ± SEM, n=3]. B) Representative H&E staining images of liver, spleen, and kidney tissues collected from mice in the control and +OSMI-1 groups. No obvious histopathological abnormalities were observed in these organs after treatment. Scale bars are 100μm and 20μm, as indicated [n=3]. C) Serum biochemical analysis of renal function-related indices, including BUN, CREA, and UA, in mice from the indicated groups. OSMI-1-1 and OSMI-1-2 represent the 10 mg/kg and 15 mg/kg dose groups, respectively. The reference ranges and measurement units are shown in the table [n=3]. D) Serum biochemical analysis of liver function-related indices, including ALT, AST, TBIL, DBIL, ALB, ALP, γ-GT, and TBA, in mice from the indicated groups. The reference ranges and measurement units are shown in the table [n=3].

**Figure S7.** A) Western blot confirmation of TJP1 protein depletion in CaKi-1 cells. B) Western blot validation of the control group, TJP1 knockdown, TJP1 rescue post-knockdown, and mutation at the TJP1-S964A O-GlcNAcylation site in CaKi-1 cells. C) Western blot validation of TJP1 knockdown and mutation at the TJP1-S964T O-GlcNAcylation site in CaKi-1 cells. D) Transwell migration/invasion assays and corresponding statistical analyses for control, TJP1-knockdown, TJP1-knockdown rescue, and TJP1-S964A O-GlcNAcylation site mutation groups [data are presented as the mean ± SEM, n=3, one-way ANOVA]. E) Transwell migration assays and corresponding statistical analyses for TJP1-knockdown rescue, TJP1-S964A O-GlcNAcylation site mutation, and TJP1-S964T O-GlcNAcylation site mutation groups [data are presented as the mean ± SEM, n=3, one-way ANOVA]. F) Transwell migration assays and corresponding statistical analyses for TJP1-knockdown and TJP1-knockdown+OSMI-1 groups [data are presented as the mean ± SEM, n=3, independent samples t-test]. G) Statistical charts of the cell aggregation assay showing cell–cell adhesion capacity in the indicated groups[data are presented as the mean ± SEM, n=3, independent samples t-test]. H) Quantification of FITC-dextran uptake/permeability in the indicated groups. TJP1 knockdown significantly increased FITC-dextran passage, whereas re-expression of TJP1 partially restored barrier function. Data are presented as violin plots [data are presented as the mean ± SEM , n=3, one-way ANOVA].

**Figure S8.** A) Histopathological examination of ccRCC across various grades: Grade Ⅰ (n=57), Grade Ⅱ (n=50), Grade Ⅲ (n=21), Grade Ⅳ (n=22), and the corresponding immunohistochemical findings and statistical evaluation [data are presented as the mean ± SEM, n=150, one-way ANOVA]. B) Quantitative proteomic analysis of primary RCC lesions and lung metastases within a murine orthotopic metastasis model [data are presented as the mean ± SEM, n=5, paired Student's t-test].
